# Mating Status of an Herbivorous Stink Bug Female Affects the Emission of Oviposition-Induced Plant Volatiles Exploited by an Egg Parasitoid

**DOI:** 10.3389/fphys.2019.00398

**Published:** 2019-04-12

**Authors:** Gianandrea Salerno, Francesca Frati, Eric Conti, Ezio Peri, Stefano Colazza, Antonino Cusumano

**Affiliations:** ^1^Dipartimento di Scienze Agrarie, Alimentari ed Ambientali, Università degli Studi di Perugia, Perugia, Italy; ^2^Dipartimento di Scienze Agrarie, Alimentari e Forestali, Universitá degli Studi di Palermo, Palermo, Italy; ^3^UMR 1333 DGIMI, INRA, Université de Montpellier, Montpellier, France

**Keywords:** *Trissolcus basalis*, *Nezara viridula*, *Vicia faba*, indirect plant defenses, OIPVs, elicitor

## Abstract

Insect parasitoids are under selection pressure to optimize their host location strategy in order to maximize fitness. In parasitoid species that develop on host eggs, one of these strategies consists in the exploitation of oviposition-induced plant volatiles (OIPVs), specific blends of volatile organic compounds released by plants in response to egg deposition by herbivorous insects. Plants can recognize insect oviposition via elicitors that trigger OIPVs, but very few elicitors have been characterized so far. In particular, the source and the nature of the elicitor responsible of egg parasitoid recruitment in the case of plants induced with oviposition by stink bugs are still unknown. In this paper, we conducted behavioral and molecular investigations to localize the source of the elicitor that attracts egg parasitoids and elucidate the role of host mating in elicitation of plant responses. We used as organism study model a tritrophic system consisting of the egg parasitoid *Trissolcus basalis*, the stink bug host *Nezara viridula* and the plant *Vicia faba*. We found that egg parasitoid attraction to plant volatiles is triggered by extracts coming from the dilated portion of the stink bug spermathecal complex. However, attraction only occurs if extracts are obtained from mated females but not from virgin ones. Egg parasitoid attraction was not observed when extracts coming from the accessory glands (mesadene and ectadene) of male hosts were applied, either alone or in combination to plants. SDS-PAGE electrophoresis correlated with olfactometer observations as the protein profile of the dilated portion of the spermathecal complex was affected by the stink bug mating status suggesting post-copulatory physiological changes in this reproductive structure. This study contributed to better understanding the host location process by egg parasitoids and laid the basis for the chemical characterization of the elicitor responsible for OIPV emission.

## Introduction

Hymenopteran parasitoids play a key ecological role in many ecosystems and these insects have been recently suggested to be the most species-rich group within the animal kingdom (Forbes et al., 2018). Many parasitoid species are natural enemies of insect pests, which can be used in integrated pest management with the aim of reducing pesticide applications (van Lenteren, 2012; Heimpel and Mills, 2017). One of the key aspects for the success of a parasitoid species as biological control agent is its host finding efficiency: parasitoids can exploit a wide array of cues when foraging for hosts, among which chemical cues convey essential information (Fatouros et al., 2008; Colazza et al., 2014). When parasitoids are far from their hosts, they can use volatile chemicals to narrow-down the area in which hosts are present, whereas contact chemicals become progressively more important once in the close proximity or in contact with the host (Vinson, 1998).

Egg parasitoids have evolved the capacity to develop exclusively on host eggs (Godfray, 1994). The ability to kill the pest before the crop feeding stage makes them very attractive in biological pest control as crop damage is kept to a minimum (Tamiru et al., 2015; Fatouros et al., 2016). Due to this adaptation, egg parasitoids face specific challenges as host eggs are generally inconspicuous and their quality decreases rapidly with egg age because of embryo development (Vinson, 1998; Fatouros et al., 2008). To cope with these challenges, egg parasitoids have evolved specific strategies in order to discover patches where host eggs have just been laid or where gravid host females are present. One of these strategies consists in the exploitation of oviposition-induced plant volatiles (OIPVs), specific blends of volatile organic compounds released by plants in response to egg deposition by herbivorous insects (Hilker and Fatouros, 2015).

Oviposition-induced plant volatiles are known to be widespread within the plant kingdom, as they have been reported to occur in several plant species, regardless of being gymnosperms or angiosperms, annuals or perennials (Fatouros et al., 2016). Depending on the egg deposition modality of the herbivores, OIPV emission can be associated with mechanical damage, as in the elm leaf beetle *Xanthogaleruca luteola* (Meiners and Hilker, 2000) and in the pine sawflies *Diprion pini* and *Neodiprion sertifer* (Hilker et al., 2002; Mumm et al., 2003), or OIPV emission can occur without obvious mechanical damage, as in the case of oviposition by butterflies and moths (Tamiru et al., 2011; Fatouros et al., 2012, Salerno et al., 2013). In the case of the stink bug *Nezara viridula*, a combination of feeding and oviposition activity is required for the emission of OIPVs that recruit egg parasitoids (Colazza et al., 2004; Moujahed et al., 2014, Frati et al., 2017).

Plants can recognize insect oviposition via elicitors that trigger OIPV release or changes in plant cuticular waxes leading to parasitoid recruitment (Hilker and Fatouros, 2015). Very few elicitors have been characterized so far but their chemical nature and origin can vary broadly. In the pine sawfly *D. pini*, the active principle attracting the egg parasitoid *Chrysonotomyia ruforum* to Scots pines is localized in the oviduct secretions and it has been shown to be of proteinaceous nature (Hilker et al., 2005). In *Pieris* butterflies, antiaphrodisiacs (benzyl cyanide and indole) transferred by the male butterfly to the female during mating induce changes in the epicuticular waxes of brassicaceous plants leading to arrestment of *Trichogramma* wasps (Fatouros et al., 2009; Blenn et al., 2012). Thus, in the latter case, it is remarkable to note that egg parasitoids gain reliable information for host location using a sophisticated strategy that involves exploitation of host sexual communication mediated by the plant.

The source and the nature of the elicitor responsible of egg parasitoid recruitment by leguminous plants induced with stink bug oviposition and feeding is still unknown. Previous studies have shown that scelionid egg parasitoids can eavesdrop the sexual communication of their stink bug hosts (Laumann et al., 2007, 2011) and distinguish between mated and virgin host adults: for example the egg parasitoid *Trissolcus basalis* searches intensively in patches contaminated with contact chemicals left by *N. viridula* host adults with a preference for those cues released by females in a pre-ovipositional state (Colazza et al., 1999). The egg parasitoid *Trissolcus brochymenae* exhibits an even more finely-tuned strategy to locate the host *Murgantia histrionica* as the wasp can discriminate between mated females that have not yet laid eggs and those that already had (Salerno et al., 2009). In particular, morphological and physiological alterations in the dilated portion of the spermathecal complex (=bursa copulatrix) after mating have been shown to be important for egg parasitoid attraction (Salerno et al., 2009). After copulation, the physiological status of females changes, with possible consequences for the chemical profile of cuticular hydrocarbons (Zahn et al., 2008), which are used by egg parasitoids to discriminate between male and female hosts (Colazza et al., 2007; Salerno et al., 2009).

In this paper, we investigated if the mating status of an herbivorous stink bug female affects the elicitation of OIPVs exploited by an egg parasitoid. In addition to sperm, stink bug males also transfer other seminal substances during mating which have been detected in female organs such as the dilated portion of the spermathecal complex and the ovary (including the common oviduct) (Koshiyama et al., 1993, Salerno and Cusumano, personal observations). These proteinaceous substances are produced in male accessory glands (ectadene and mesadene) and have been suggested to have nutritional functions for the females (McLain, 1980; Kon et al., 1993). Using as model study organisms the tritrophic system consisting of the egg parasitoid *T. basalis*, the host *N. viridula* and the plant *Vicia faba*, we conducted behavioral and molecular investigations to localize the source of the elicitor that attracts egg parasitoids and elucidate the role of host mating in elicitation of plant responses. We tested two alternative hypothesis: (1) substances transferred from host males to females during mating could be directly implicated in the attraction of the egg parasitoid toward OIPVs; (2) mating induces physiological changes in host females that indirectly trigger OIPV emission and egg parasitoid attraction.

## Materials and Methods

### Insects and Plants

The *N. viridula* colony, established from material collected in cultivated and uncultivated fields around Perugia, was reared under controlled conditions (24 ± 2°C; 70 ± 5% RH;16 h:8 h L:D) inside clear plastic food containers (30 cm × 19.5 cm × 12.5 cm) with 5 cm diameter mesh-covered holes for ventilation. Bugs were fed with a diet of sunflower seeds and seasonal fresh vegetables. Food was changed every 2–3 days, and separate cages were used for nymphs and adults. Egg masses were collected daily and used to maintain colonies of both *N. viridula* and *T. basalis.* The *N. viridula* colony was supplemented regularly with field-collected bugs.

The colony of *T. basalis* was originally established from wasps emerging from *N. viridula* egg masses, located in wild and cultivated fields around Perugia. The parasitoid was reared on *N. viridula* egg masses that were glued on paper strips. Wasps were maintained in 85 ml glass tubes, fed with a honey-water solution and kept in controlled climatic chamber under the same rearing conditions of *N. viridula*. After emergence, male and female wasps were kept together to allow mating. For all bioassays, 2–4 days old females. All tested wasps were naïve (i.e., they had no previous contact with plants).

Seeds of broad bean plants (*V. faba* cv. Superaguadulce) were individually planted in plastic pots (9 cm × 9 cm × 13 cm) filled with a mixture of agriperlite (Superlite, Gyproc Saint-Gobain, PPC Italia, Italy), vermiculite (Silver, Gyproc Saint-Gobain, PPC Italia, Italy), and sand (1:1:1) and grown in a climate controlled chamber (24 ± 2°C, 45 ± 10%RH, 12 h:12 h L:D). Plants were watered daily and, from 1 week post-germination, fertilized with an aqueous solution (1.4 g/l) of fertilizer (5-15-45, N-P-K, Plantfol, Valagro, Italy). Plants with approximately 4 expanded leaves were used for experiments.

### Treatments

**FIGURE 1:**
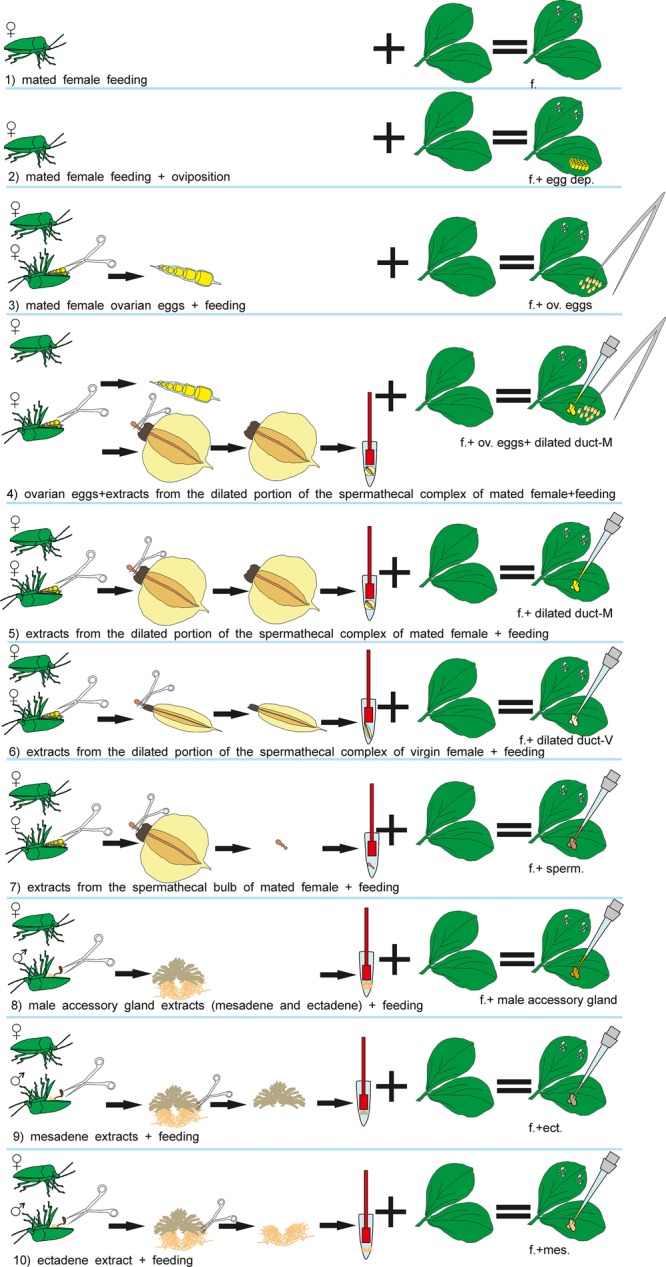
Visual summary of *Nezara viridula* dissections and subsequent treatments of *Vicia faba* plants.

The test plants used in the bioassays were always exposed to *N. viridula* females and subjected to the following treatments (Figure 1): (1) feeding; (2) feeding + oviposition; (3) ovarian eggs + feeding; (4) ovarian eggs + extracts from the dilated portion of the spermathecal complex of mated females + feeding; (5) extracts from the dilated portion of the spermathecal complex of mated females + feeding; (6) extracts from the dilated portion of the spermathecal complex of virgin females + feeding; (7) extracts from the spermathecal bulb + feeding; (8) male accessory gland extracts (mesadene and ectadene) + feeding; (9) mesadene extracts + feeding; (10) ectadene extract + feeding. Previous studies have shown that parasitoid attraction is not observed when plants are induced with eggs only leading to the exclusion of egg deposition treatment from the current study (Colazza et al., 2004). Similarly, previous works have shown that OIPV recruiting egg parasitoids are emitted only when egg deposition occurs in combination with feeding activity of stink bugs (Colazza et al., 2004); thus we decided to include stink bug feeding activity in all the treatments.

For all treatments (1–10), a mated *N. viridula* female was placed for 24 h on the lower surface of a leaf in the central plant portion, inside a “clip cage” made of two modified plastic Petri dishes (Ø = 10 cm; *h* = 1 cm), with a mesh- covered hole in the bottom and the rim covered by a small sponge ring. This plant–bug complex was kept in a controlled environment cabinet (24 ± 2°C, 55 ± 10% RH; 12 h:12 h L:D) allowing the insect to feed and oviposit (treatment 2) or to feed (treatments 1,3-10) and after 24 h the insect was removed. For treatments 1,3-10, plants where the females oviposited were discarded. For treatments 3–10 ovarian eggs and/or extracts were applied together with the insect exposure. Treated plants were used for all the bioassays 24h after the end of the insect exposure. Healthy plants with an empty clip cage applied on a leaf for 24h, were used as controls (24 h after the clip removal).

To exclude an effect of the glue that stink bug females use to attach eggs on the leaves, plants with ovarian eggs were used in treatments 3 and 4. Plants with ovarian eggs (treatment 3) were obtained by applying carefully with a tiny brush 30 ovarian eggs near the clip cage confining the *N. viridula* female onto the lower leaf surface. Ovarian eggs were collected by dissecting *N. viridula* mated females. Females were previously anesthetized at -4°C for 3 min and dissected with the aid of micro-scissors in phosphate buffered saline solution (PBS).

To obtain plants treated with extracts from the spermathecal complex of females and from male accessory glands (treatments 4–10), stink bugs were anesthetized and dissected with the aid of micro-scissors as described above. From stink bug females, the spermathecal apparatus was removed from the abdomen and separated in: (a) the dilated portion of the spermathecal complex and (b) the spermathecal bulb, which were placed in different 1.5 ml Eppendorf tubes containing 10 μl of PBS each (one organ/tube). Tissues were disrupted with the aid of a pestle, then centrifuged at 10000 × *g* for 5 min at 4°C and finally 10 μl of supernatant (=1 bug equivalent) was collected from each sample to be used for plant induction. Stink bug males were anesthetized and dissected similarly to females, and male accessory glands (mesadene and ectadene) were isolated using micro-scissors and placed separately in Eppendorf tubes containing 10 μl of PBS (one organ/tube). Tissues were disrupted, centrifuged at the same conditions as described for females, and 10 μl of supernatant was collected.

In the treatments 4–7, plants were induced with extracts obtained from female stink bugs. In particular, for treatment 4, 30 ovarian eggs were placed into contact with the extract from the spermathecal complex for 3 min and then eggs were carefully applied to a plant leaf with a brush as described above. 10 μl of extract obtained from the dilated duct of the spermathecal complex of mated or virgin females was applied directly onto the leaf in treatments 5 and 6, respectively. For treatment 7, 10 μl of extract obtained from the spermathecal bulb of mated females was applied onto the leaf of one plant.

In the treatments 8–10, plants were treated with extract obtained from accessory glands of virgin males (mesadene and ectadene). One bug equivalent (10 μl) of each gland was used for treatment 8 whereas mesadene and ectadene extracts were used separately to induce plants for treatment 9 and treatment 10 respectively.

### Y-Tube Bioassays

Wasps’ responses to volatile chemicals from differently treated *V. faba* plants were investigated with a dual choice Y-tube olfactometer made from a polycarbonate body (stem 9 cm; arms 8 cm at 130° angle; ID 1.5 cm) sandwiched between two glass plates. A stream of clean air (medical-grade compressed air, N_2_:O_2_ 80:20), humidified by bubbling through a water jar, was regulated in each arm by a flowmeter at about 0.40 l min^-1^. The device was illuminated from above by two 22-W cool white fluorescent tubes, and from below by an infrared source (homogeneous emission of wavelengths at 950 nm provided by 108 LEDs). Before entering the olfactometer arms, each airstream passed through a cylindrical glass chamber (Ø = 12 cm; *h* = 52 cm) with an O-ring sealed middle joint, containing a treated plant as odor source. The stimuli were randomly assigned at the beginning of the bioassays and were switched regularly to avoid possible bias in the system. At every switch, the whole system was changed with clean parts. At the end of the bioassays, the polycarbonate olfactometer and all glass parts were cleaned with water. The glass parts were then cleaned with acetone and baked overnight at 180°C. Wasp females were singly introduced into the Y-tube olfactometer at the entrance of the stem and allowed to move freely for 10 min. Their behavior was recorded using a monochrome CCD video camera (Sony SSC M370 CE) fitted with a 12.5–75 mm/F 1.8 zoom lens. The camera lens was covered with an infrared pass filter (Kodak Wratten filter 87 Å) to remove visible wavelengths. Analog video signals from the camera were digitized by a video frame grabber (Canopus^®^ ADVC 110, Grass Valley CA, United States).

**FIGURE 2:**
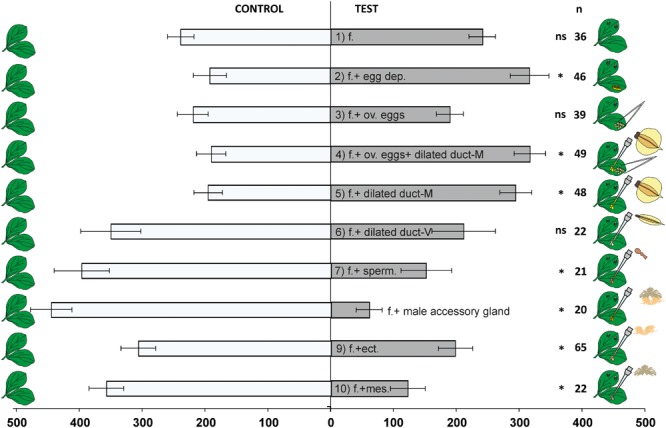
Response of *Trissolcus basalis* females in a Y-tube olfactometer to volatiles from *V. faba* plants subjected to different treatments *versus* healthy plants (used as controls). f. = feeding; egg dep. = oviposition; ov. eggs = ovarian eggs; dilated duct-M = dilated portion of the spermathecal complex in mated females; dilated duct-V = dilated portion of the spermatechal complex in virgin females; sperm. = spermathecal bulb, mes. = mesadene, ect. = ectadene. N = number of replicates. Bars represent mean (±SE) of the time spent by wasp females in each arm over an observation period of 600 s (*t*-test for dependent samples, ns = not significant; ^∗^*P* < 0.05).

Digitized data were processed by XBug, a video tracking and motion analysis software (Colazza et al., 1999). Wasp response was measured in terms of residence time, i.e., the time spent by the wasps in each arm during the entire bioassay. The Y-tube olfactometer bioassays were carried out as paired choices, in which plants treated as described in the previous section were always tested *versus* healthy plants used as control. For each paired combination at least 20 replicates have been carried out (see Figure 2).

### Protein Concentration and SDS-PAGE

To investigate changes in morphology and protein content occurring in the dilated portion of the spermathecal complex after mating, virgin and mated females were dissected as described above and for each female we quantified: (1) the area of the dilated portion of the spermathecal complex measured under an Olympus MVX10 stereoscope fitted with a XC50 camera using the Olympus cellSens digital imaging software; (2) the protein concentration in the dilated portion using the Bradford bioassays (Bradford, 1976). For each group, 6–7 replicates were carried out.

SDS-PAGE gel electrophoresis was carried out to compare the protein profiles of male accessory glands (ectadene and mesadene) with the profile of the dilated portion of the spermathecal complex in females (both virgin and mated ones). For each of the four groups, gland extracts were obtained as described in the previous section and samples containing 20 μg of proteins, derived by pooling together 5 insects, were loaded in each lane. The proteins in the samples were separated using Precast 4–15% Ready Gel (Bio-Rad), under denaturating conditions. Electrophoresis was performed in 25 mM Tris-HCl pH 8.8, 195 mM glycine, and 0.1% (w/v) SDS at a constant current of 30 mA. Gels were stained with Coomassie Brilliant blue R-250 (Bio-Rad). Preliminary SDS-PAGE electrophoresis loading lanes with extracts from single spermathecal organs were replicated 3–4 times for virgin and mated females, respectively, to evaluate the consistency of protein profile patterns (see Supplementary Figure 1).

### Statistical Analyses

For the olfactometer bioassays, data was tested for normality (Shapiro-Wilk test). Because there was no significant deviation from normal distribution, the time spent by wasp females in each arm was statistically compared by parametric paired *t*-tests for dependent samples. Linear models were used to test the effect of mating status on the protein concentration of the dilated portion of the spermathecal complex, using the area of the dilated portion as covariate in the model. Significance of the variables in the model was determined using Likelihood Ratio Tests (LRTs) comparing the full model with and without the variable in question starting with interaction effects (Crawley, 2007). Model fit was assessed with residual plots. SDS-PAGE gel obtained by loading lanes with extracts from single spermathecal organs of virgin and mated females were analyzed with the software program Scion Image (Scion Corp., Frederick, MY, United States) in order to assess protein amounts. The detailed steps for relative quantification of band proteins can be found in the software manual^1^. Relative areas of the most characteristic bands associated with virgin and mated females were compared with *t*-tests after checking that assumptions of normality were met (Shapiro-Wilk test). Olfactometer data were analyzed using the STATISTICA7 software (StatSoft Inc., 2001) whereas all other analyses were carried out using the R software version 3.4.4 (R Core Team, 2013).

## Results

### Olfactometer Bioassays

*Trissolcus basalis* females were significantly attracted to volatiles emitted by plants induced with a combination of feeding and oviposition activity of *N. viridula* (*t* = -2.10, df = 45, *P =* 0.0411) (Figure 2). On the contrary, wasps did not prefer plants induced only with stink bug feeding activity when tested against control plants (*t* = -0.10, df = 35, *P =* 0.9203). Similarly, the addition of ovarian eggs to plants damaged by stink bug feeding did not trigger parasitoid attraction (*t* = 0.99, df = 38, *P =* 0.3265). Regardless of the application of ovarian eggs, *T. basalis* attraction was restored when extracts coming from the dilated portion of the spermathecal complex of mated females were applied to plants (ovarian eggs + feeding + spermathecal extracts: *t* = 2.73, df = 48, *P =* 0.0088; feeding + spermathecal extracts: *t* = 2.22, df = 47, *P =* 0.0315). On the contrary, extracts coming from the dilated portion of the spermathecal complex of virgin females did not trigger parasitoid attraction when tested over control plants (*t* = -1.43, df = 21, *P =* 0.1670). Parasitoids preferred the control plants when tested against *N. viridula* feeding-induced plants which were treated with extracts coming from the spermathecal bulb (*t* = -2.76, df = 20, *P =* 0.0121). A similar preference for control plants was observed when extracts coming from the male accessory glands (mesadene and ectadene) were applied, either alone (ectadene: *t* = -2.09, df = 64, *P =* 0.0409; mesadene: *t* = -4.59, df = 21, *P =* 0.0002) or in combination (*t* = -7.30, df = 19, *P <* 0.0001) to plants damaged by *N. viridula* feeding activity.

### Protein Concentration and SDS-PAGE

**FIGURE 3:**
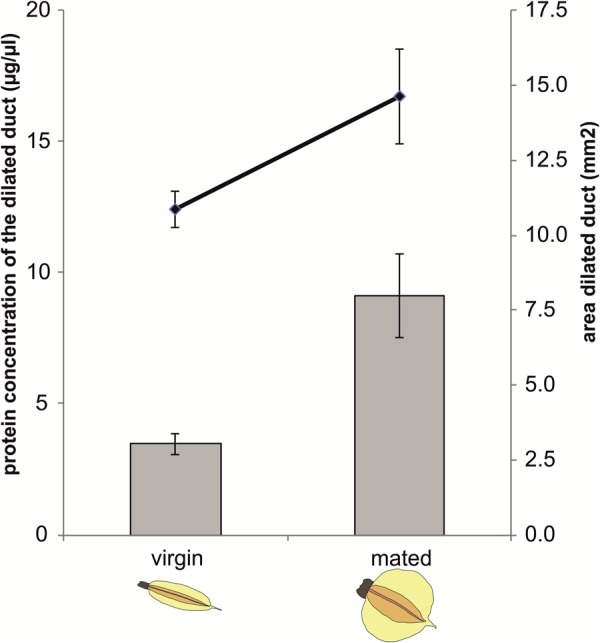
Protein concentration and size of the dilated portion of the spermathecal complex in virgin and mated *Nezara viridula* females. Bars represent means ± SE of protein concentrations (μg/μl) in virgin and mated bugs. Lines represent means ± SE of the size of the organs (mm^2^) in virgin and mated bugs (GLM, ^∗^*P* < 0.05). For each group, 6–7 replicates were carried out.

Protein concentration in the dilated portion of the spermathecal complex was significantly affected by mating status (GLM: χ^2^= 4.96, df = 1, *P =* 0.0259) as well as by the area of the organ (χ^2^= 14.25, df = 1, *P =* 0.0002) whereas there was no significant mating status × area interaction (χ^2^= 0.71, df = 1, *P =* 0.3979) (Figure 3).

**FIGURE 4:**
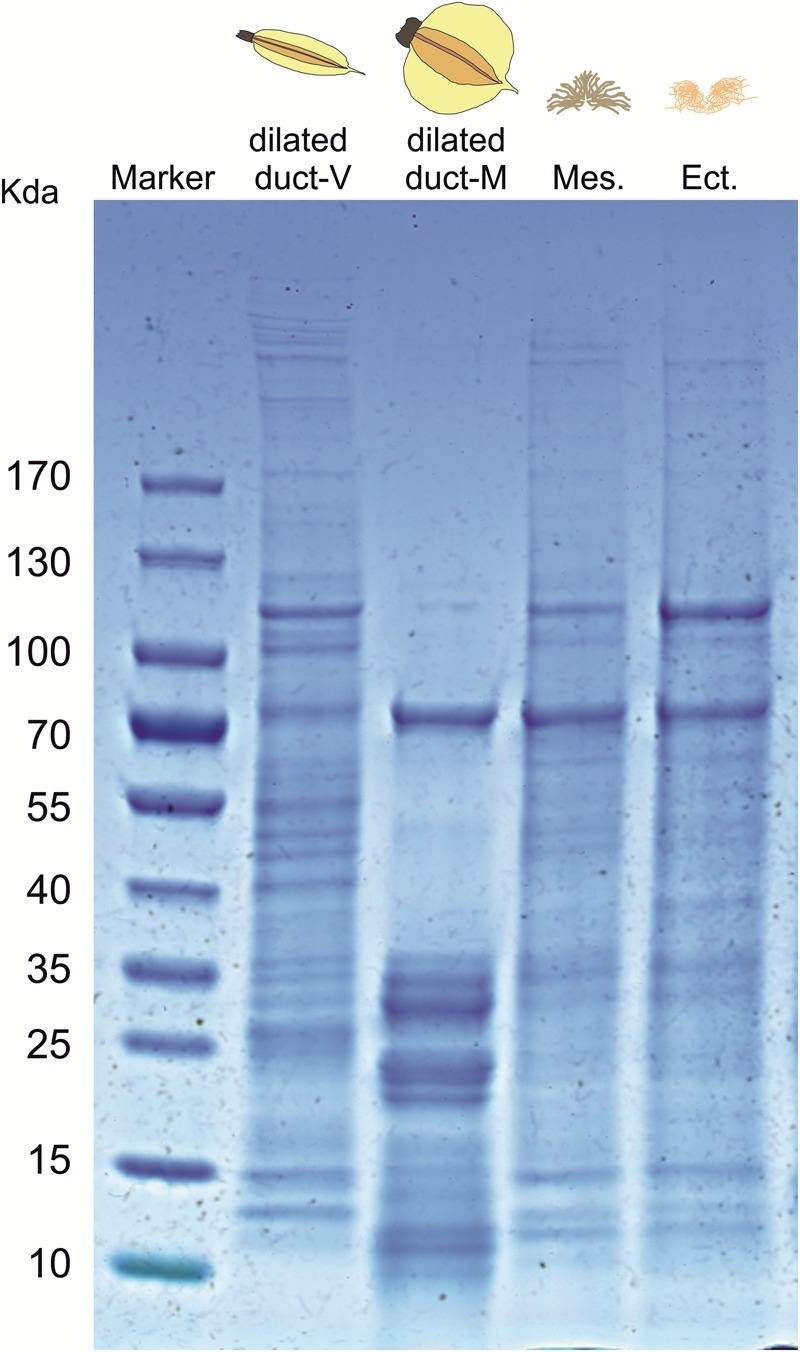
SDS-PAGE profile of: the dilated portion of the spermathecal complex in virgin (V) and mated (M) *Nezara viridula* females; male accessory gland mesadene (Mes) and ectadene (Ect). Each lane was loaded with biological material derived by pooling five stink bug insects. An equivalent lane from the same gel (4–15% SDS-PAGE) showing the molecular mass of the protein marker is presented on the left stained with Coomassie Brilliant blue R-250.

The SDS-PAGE profile of the dilated portion of the spermathecal complex consists of several proteins ranging in size from ∼10 to ∼170 kDa (Figure 4). The protein profile appears to be different between virgin and mated females although we cannot exclude the possibility that protein profiles simply differ in expression levels. Consistency of the whole protein profiles was observed among replicates of individual mated females (*N* = 4) as well as among virgin females (*N* = 3) (Supplementary Figure 1). Relative quantification of protein amount is different in characteristic bands associated with mated females (band n. 3 at ∼70 kDa, bands n. 8–9 at ∼35 kDa) or virgin females (bands 4–7 between 40 and 55 kDa). Some bands appear to be present only in mated (band n. 10 at ∼10 kDa) or virgin females (band n. 1–2 at ∼100 kDa) (Supplementary Table 1). The protein profile of male accessory glands (mesadene and ectadene) is very similar between them but no analogous similarity is found in terms of protein content between male accessory glands and the dilated portion of the spermathecal complex of mated females, except for a band of ∼70 kDa which deserves to be better investigated.

## Discussion

In this paper we show that mating status of the stink bug host affects the emission of OIPVs in leguminous plants leading to egg parasitoid response. Previous investigations have shown that *N. viridula* feeding and oviposition activities are required to induce *V. faba* plant volatiles that are attractive for *T. basalis* (Colazza et al., 2004; Moujahed et al., 2014, Frati et al., 2017). Application of ovarian eggs does not mimic the effect of oviposition in terms of parasitoid recruitment unless secretions belonging to the dilated portion of the spermathecal complex are also applied onto plant leaves. Interestingly, these secretions trigger wasp attraction even without the concurrent application of host eggs on the plant: however, the parasitoid response occurs only when stink bugs are mated, which suggests that the role of the dilated portion of the spermathecal complex as a source of the elicitor of OIPVs is strictly dependent on the host mating status.

In another tritrophic system it was shown that, during mating, male butterfly transfers antiaphrodisiacs (benzyl cyanide and indole) to the female butterfly along with sperm, to reduce attractiveness toward conspecific males (Andersson et al., 2003). Traces of antiaphrodisiacs were detected into female accessory glands and, when in contact with brassicaceous plants, these compounds induce wax epicuticular changes that arrest *Trichogramma* egg parasitoids (Fatouros et al., 2009). However, application of antiaphrodisiacs do not fully mimic egg deposition in terms of plant defences, as these compounds do not trigger OIPVs attracting *Trichogramma* parasitoids (Fatouros et al., 2015). This is similar to our findings, as *T. basalis* attraction could not be restored when secretions from *N. viridula* male accessory glands were applied to *V. faba* plants highlighting the intricacy of plant responses to insect egg deposition. In our model-study system we found that parasitoids were repelled when extracts from mesadene and ectadene were applied alone or in combination to the plants. As a similar effect was also found when plants were treated with spermathecal bulb extracts containing sperm, it is possible to argue that the elicitor is not a male-derived substance(s) capable *alone* to trigger in the plant the emission of volatiles that attract egg parasitoids. Whereas it is difficult to interpret these repellence effects in the light of the foraging strategy of egg parasitoids, these results confirmed the importance of mating for the elicitation of OIPVs, suggesting that female-derived substances are also needed for restoring parasitoid attraction.

In several heteropteran species, mating is a complex process that can last several days during which sperm and associated seminal fluids are transferred from males to females (Schrader, 1960; Mitchell and Mau, 1969; Mau and Mitchell, 1978; Kasule, 1986; Koshiyama et al., 1993; Wang and Millar, 1997; Ho and Millar, 2001a,b). Because seminal fluid proteins are known to be transferred during mating in *N. viridula* (Kon et al., 1993; Koshiyama et al., 1993), we investigated the protein profiles of male accessory glands and of the dilated portion of the spermathecal complex (in both virgin and mated females) to look for pattern similarities. SDS-PAGE results suggest that the protein profile of the dilated portion of the spermathecal complex is affected by the insect mating status although expression studies are needed to confirm increased abundance (a band at ∼70 kDa, and multiple bands at ∼35 kDa) or presence of specific proteins (a band at ∼10 kDa) only in mated females. This finding correlated with the behavioral assays, since only secretions from *N. viridula* mated bugs elicit *T. basalis* attraction to *V. faba* plant volatiles. Nonetheless, additional investigations are clearly required to provide evidence for the proteinaceous nature of the elicitor but it should be noted that the only identified elicitors of OIPV release and parasitoid recruitment are small proteinaceous compounds (Hilker et al., 2005). In addition, SDS-PAGE indicated no clear similarity of the protein patterns among male accessory glands and the dilated portion of the spermathecal complex of mated females, except for a band of ∼70 kDa which should be investigated with expression studies.

Here we propose two possible scenarios to explain our results. (1) Mating event triggers changes in the physiological status of the females, which, in turn, are *indirectly* responsible of egg parasitoid recruitment. In fact, it is known that seminal fluid proteins produced in reproductive tract tissues of male insects and transferred to females during mating induce numerous physiological and behavioral post-mating changes in females (Gillott, 2003; Avila et al., 2011). The fact that, according to our SDS-PAGE results, some proteins seem to be only present in the dilated portion of the spermathecal complex of virgin females (and disappear in mated ones) could be a further indication of the complexity of these post-mating changes. Thus, in our study, physiological changes stimulated by seminal fluids in *N. viridula* could trigger OIPV emission in *V. faba* and *T. basalis* attraction. (2) Alternatively, compounds transferred by males to females during mating (i.e., a putative protein of ∼70 kDa) are *directly* responsible of egg parasitoid recruitment only in the presence of a “suitable chemical background” provided by the females. In fact, extracts of male accessory glands do not elicit an attraction response in the wasp, and activity is triggered only by the dilated portion of the spermathecal complex of mated females. Because SDS-PAGE suggests presence of a protein of ∼70 kDa that seems to be shared both in male accessory glands and in the bursa copulatrix of mated females, further investigations should be carried out in order to confirm that this protein is transferred during copulation and whether it is involved in elicitation of plant responses. Although we are not aware of any study showing activity of a putative elicitor only when in combination with the “right chemical mixture,” the importance of the background composition has been demonstrated in the chemical ecology of plant volatiles recruiting egg parasitoids. In fact, for the egg parasitoid *C. ruforum*, the key compound (*E*)-β-farnesene is only attractive when present in a contrasting background odor from undamaged pine twigs (Mumm and Hilker, 2005).

To conclude here we report that mating of *N. viridula* is essential to trigger, in *V. faba* plants, emission of volatiles that attract the parasitoid *T. basalis*. Our behavioral assays provide evidence that the source of the elicitor is localized in the dilated portion of spermathecal complex of mated *N. viridula* females and our SDS-PAGE investigations indicate that mating affects the whole protein profile of this organ. While the behavioral results correlate with changes in the dilated portion of the spermathecal complex of virgin and mated stink bug females, further behavioral assays are needed to clarify the chemical nature of the elicitor responsible of plant-mediated parasitoid attraction. In particular, treatments with proteinases are required to test if the activity of secretions coming from the dilated spermathecal duct of mated females is lost after enzymatic digestion. If the proteinaceous nature of the elicitor is confirmed, then a combined transcriptomic and proteomic approach targeting male accessory glands and the dilated portion of the spermathecal complex of virgin and mated females is needed for the chemical characterization of the proteins. A transcriptomic analysis would also allow to identify candidate genes whose function could be further studied, for example using the RNA interference technology which is known to be efficient in true bugs.

## Ethics Statement

The study used insects, but none of the manipulations involved in experiments raise ethical issues. All animal care and experimentation complied with the guidelines provided by the Association for the Study of Animal Behavior (ASAB) and the Animal Behavior Society (ABS).

## Author Contributions

All authors conceived and designed the experiments, interpreted the results, and drafted and revised the article. GS, FF, and AC performed the experiments and analyzed the data.

## Conflict of Interest Statement

The authors declare that the research was conducted in the absence of any commercial or financial relationships that could be construed as a potential conflict of interest.

## Footnotes

1 http://mesonpi.cat.cbpf.br/e2002/cursos/NotasAula/ScnImage.pdf
