# Supplementary material for: Mating Status of an Herbivorous Stink Bug Female Affects the Emission of Oviposition-Induced Plant Volatiles Exploited by an Egg Parasitoid
Source: Front Physiol. 2019 Apr 12;10:398. doi: 10.3389/fphys.2019.00398 (PMC6473057; doi:10.3389/fphys.2019.00398)
Supplement: Supplementary file 1 [file Table_1.DOCX]

**Supplementary Figure 1:** SDS-PAGE profile of the dilated portion of the spermathecal complex in mated (M-1,2,3,4) and virgin (V-1,2,3) *Nezara viridula* females. Each lane was loaded with biological material derived by a single stink bug insect. Asterisks indicate characteristic bands associated with the dilated portion of the spermathecal complex in mated (red asterisks) or virgin (blue asterisks) females. An equivalent lane from the same gel (4-15% SDS-PAGE) showing the molecular mass of the protein marker is presented on the left stained with Coomassie Brillant blue R-250. Bands highlighted with arrows are quantified in the table 1.


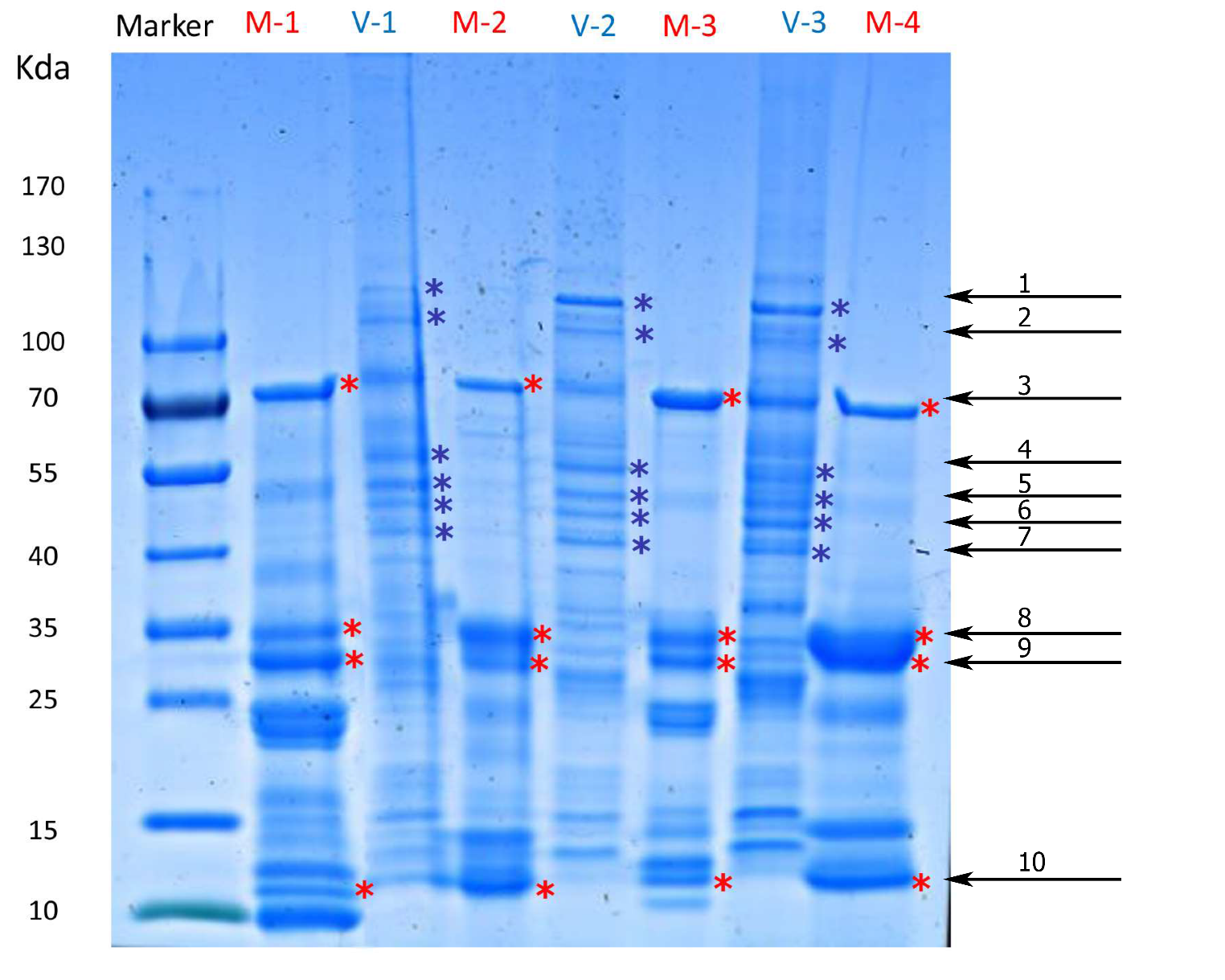


**Supplementary Table 1.** Protein amounts of 10 characteristic bands found in the SDS-PAGE gel obtained by loading lanes with extracts from single spermathecal organs of virgin and mated females. Relative quantification of band proteins is estimated with the software program Scion Image. Relative areas (in square units) of the bands were compared between virgin and mated females using *t*-tests

| **ID band** | **Virgin females**  **Area (square units) (N=3)** | **Mated females**  **Area (square units) (N=4)^#^** | ***t*-test** | | | | |
| --- | --- | --- | --- | --- | --- | --- | --- |
|  |  |  | ***t*-value** | | **df** | | ***P*** |
| 1^*^ | 248.00 ± 45.76 | 0.00 | - |  | |  | |
| 2^*^ | 210.69 ± 15.62 | 0.00 | - |  | |  | |
| 3 | 165.35 ± 54.58 | 542.54 ± 55.24 | -4.73 | 5 | | 0.0052 | |
| 4 | 213.65 ± 5.81 | 43.23 ± 8.32 | 15.52 | 5 | | <0.001 | |
| 5 | 256.74 ± 3.52 | 96.45 ± 8.10 | 14.91 | 5 | | <0.001 | |
| 6 | 248.67 ± 12.99 | 26.05 ± 3.13 | 19.39 | 5 | | <0.001 | |
| 7 | 256.01 ± 8.96 | 30.29 ± 5.61 | 22.53 | 5 | | <0.001 | |
| 8 | 59.43 ± 22.43 | 486.57 ± 62.82 | -6.41 | 4 | | 0.0031 | |
| 9 | 65.03 ± 24.58 | 570.78 ± 102.72 | -4.79 | 4 | | 0.0087 | |
| 10^*^ | 0.00 | 367.79 ± 76.61 | - |  | |  | |

^*^=qualitative differences were found between virgin and mated females. As a consequence, no *t*-test was performed for bands 1,2 and 10

^#^=for bands 8 and 9 average values and SE are calculated based on 3 samples
